# Higher dietary magnesium and potassium intake are associated with lower body fat in people with impaired glucose tolerance

**DOI:** 10.3389/fnut.2023.1169705

**Published:** 2023-04-17

**Authors:** Natural Chu, Tsz Yeung Chan, Yuen Kiu Chu, James Ling, Jie He, Kathy Leung, Ronald C. W. Ma, Juliana C. N. Chan, Elaine Chow

**Affiliations:** ^1^Department of Medicine and Therapeutics, The Chinese University of Hong Kong, Prince of Wales Hospital, Sha Tin, Hong Kong SAR, China; ^2^Department of Life Sciences, The Chinese University of Hong Kong, Sha Tin, Hong Kong SAR, China; ^3^Li Ka Shing Institute of Health Sciences, The Chinese University of Hong Kong, Prince of Wales Hospital, Sha Tin, Hong Kong SAR, China

**Keywords:** diabetes, minerals, impaired glucose tolerance, obesity, body fat, potassium, magnesium, body fat %

## Abstract

**Introduction:**

Obesity and diabetes are public health concerns worldwide, but few studies have examined the habitual intake of minerals on body composition in people with prediabetes.

**Methods:**

In this prospective cross-sectional study, 155 Chinese subjects with IGT [median age: 59 (53–62) years, 58% female] had an assessment of body composition including body fat percentage, oral glucose tolerance tests (OGTT), Homeostatic Model Assessment of Insulin Resistance (HOMA-IR) and 3-day food records from nutritional programme analysis.

**Results:**

Dietary intake of minerals was negatively correlated with body fat. People with obesity had the lowest daily consumption of iron median (IQR) 10.3 (6.9–13.3) mg, magnesium 224 (181–282) mg, and potassium 1973 (1563–2,357) mg when compared to overweight [10.5 (8.0–14.5) mg, 273 (221–335) mg, and 2,204 (1720–2,650) mg] and normal weight individuals [13.2 (10.0–18.6) mg, 313 (243–368) mg, and 2,295 (1833–3,037) mg] (*p* = 0.008, <0.0001, and 0.013 respectively). Amongst targeted minerals, higher dietary magnesium and potassium intake remained significantly associated with lower body fat after the adjustment of age, gender, macronutrients, fibre, and physical activity.

**Conclusion:**

Dietary magnesium and potassium intake may be associated with lower body fat in people with impaired glucose tolerance. Inadequate dietary mineral intake may play contribute to obesity and metabolic disorders independent of macronutrients and fibre consumption.

## Introduction

Lifestyle modification is an integral component of the management of diabetes and cardiometabolic disorders. Dietary intervention improves glycaemic control and insulin resistance with 3–5% of weight loss in subjects with overweight and obese, resulting in decreased mortality and morbidity associated with type 2 diabetes (T2D) (1). Apart from prioritising reduction in the intake of energy-dense foods, a balanced diet with the consumption of whole grains, vegetables, and fruits is also important. However, current dietary interventions predominantly focus on altered macronutrient intake, with less focus on regulating micronutrient intake.

Body fat accumulation is the single-most important risk factor for metabolic syndrome and its predisposition to diabetes (2). Lifestyle factors contributing to the former include inadequate physical activity and overnutrition, such as high intake of refined carbohydrates (3). Additionally, it can result in an inadequate intake of essential vitamins and minerals (4, 5). The relationship between mineral intake and impaired glucose tolerance (IGT) is of particular interest, given the known roles of minerals such as magnesium in improving glucose, insulin, and lipid metabolism (6). Subjects with IGT have a decompensation rate of 1–10% per year, with a cumulative incidence of T2D as high as half over time (7, 8).

A nutritional survey using 24 h dietary recall showed that higher dietary magnesium was associated with lower body mass index (BMI) in the Mexican cohort without a known diagnosis of diabetes (9), but inconsistent results were observed using food frequency questionnaires (FFQ) in patients with diabetes (10). Moreover, dietary sodium and potassium play a pivotal role in blood pressure regulation and intracellular osmolarity (11, 12). Moreover, dietary potassium was associated with obesity and metabolic syndrome (13). Few studies have investigated detailed relationships between dietary minerals and body fat composition in people with prediabetes which may also influence response to intensive dietary interventions. Furthermore, most studies used food frequency questionnaires (FFQ) or 24-h recall to capture habitual intake which may provide inaccurate data due to recall bias, retrospective and incomplete records (14). The study aimed to investigate the relationship between dietary minerals, body composition, and metabolic parameters in subjects with impaired glucose tolerance using accurate diagnosed methods (OGTT) and prospective food records. Therefore, in this study, we examined the associations between habitual minerals and anthropometric, biochemical parameters, and glycemic response in subjects with impaired glucose tolerance (IGT) using prospective 3-day food records.

## Methods

Participants were recruited as part of screening for randomised controlled trial for continuous glucose monitoring (CGM) as an adjunct to lifestyle modification in IGT (NCT04588896). Participants were identified from those attending the medical outpatients at Prince of Wales Hospital, general outpatient clinics in the New Territories East Cluster, or self-referral from the community in Hong Kong SAR. We recruited participants above 18 years of age and <65 years old, BMI of 18–40 kg/m^2^, not pregnant or lactating, with no history of diabetes and treatment with glucose-lowering drugs or any weight-loss treatment. Those who were participating in weight loss interventions within 3 months of the screening period were excluded. All participants underwent a 75 g OGTT was performed after an overnight fast. Glycaemic status and T2D were defined according to the ADA criteria (2009) for impaired glucose tolerance (IGT): the range of 2-h glucose level was between 7.8 mmol/L and < 11.1 mmol/L.

### Dietary evaluation

This is a prospective evaluation of dietary intake using food records over a three-day period to reflect habitual consumption (records two weekdays and one weekend day). In a food record, the subject records all the food and beverages consumed, including ingredients, cooking method, and quantity of the food consumed at a given period. Upon return of the food records, a dietitian carefully screened the records according to standardised portion sizes and clarified any missing information with the patient. Food records were then analysed for daily intake of 8 targeted minerals: calcium, chromium, iron, magnesium, phosphorous, potassium, selenium, and sodium using a nutritional analysis programme (eSHA Food Processor Nutrition Analysis Software). The sodium-potassium ratio is the amount of sodium compared to potassium in the diet.

### Anthropometric and biochemical measurements

Body fat percentage was assessed by an impedance biochemical analysis system [Tanita; Model: TBF-410 Body composition analyser; Nagai et al., 2008 (15); Thomas et al., 2010 (16)]. Waist and hip circumference (cm) were measured at the baseline. Height was measured with a stadiometer to the nearest 0.1 cm for the calculation of BMI. Moreover, we categorised the body weight into three groups under the World Health Organization (WHO) Asian classification for obesity (17), normal weight is between 18 and 22.9 kg/m^2^, overweight is 23–26.9 kg/m^2^, and obesity is higher or equal than 27 kg/m^2^. Physical and activity levels were recorded using the International Physical Activity Questionnaires (IPAQ) (Chinese version) (18).

Fasting blood samples were collected following an 8-h overnight fast. Venous blood samples were collected from participants in a six-point 75 g oral glucose tolerance test (OGTT) at 0, 15, 30, 60, 90, and 120 min with C-peptide measurement. Homeostasis Model Assessment calculator (HOMA2) was used to estimate HOMA-IR and HOMA-β indices as percentages of a normal reference population developed by Oxford University (version 2.2.4 Diabetes Trials Unit, University of Oxford, Oxford, United Kingdom) (19).

### Statistical analysis

Statistical analysis of all data was performed using SPSS version 26.0 (SPSS Inc., Chicago, IL, United States). To investigate the associations between the investigated minerals and markers of glucose-dependent parameters, multivariate analyses were conducted amongst the study cohort. Data were presented as a β coefficient, *p*-value, and 95% confidence interval. Values were reported as mean ± SD for parametric data or median [interquartile range] for non-parametric data. Significance was set at *p* ≤ 0.05.

## Results

**Figure 1:**
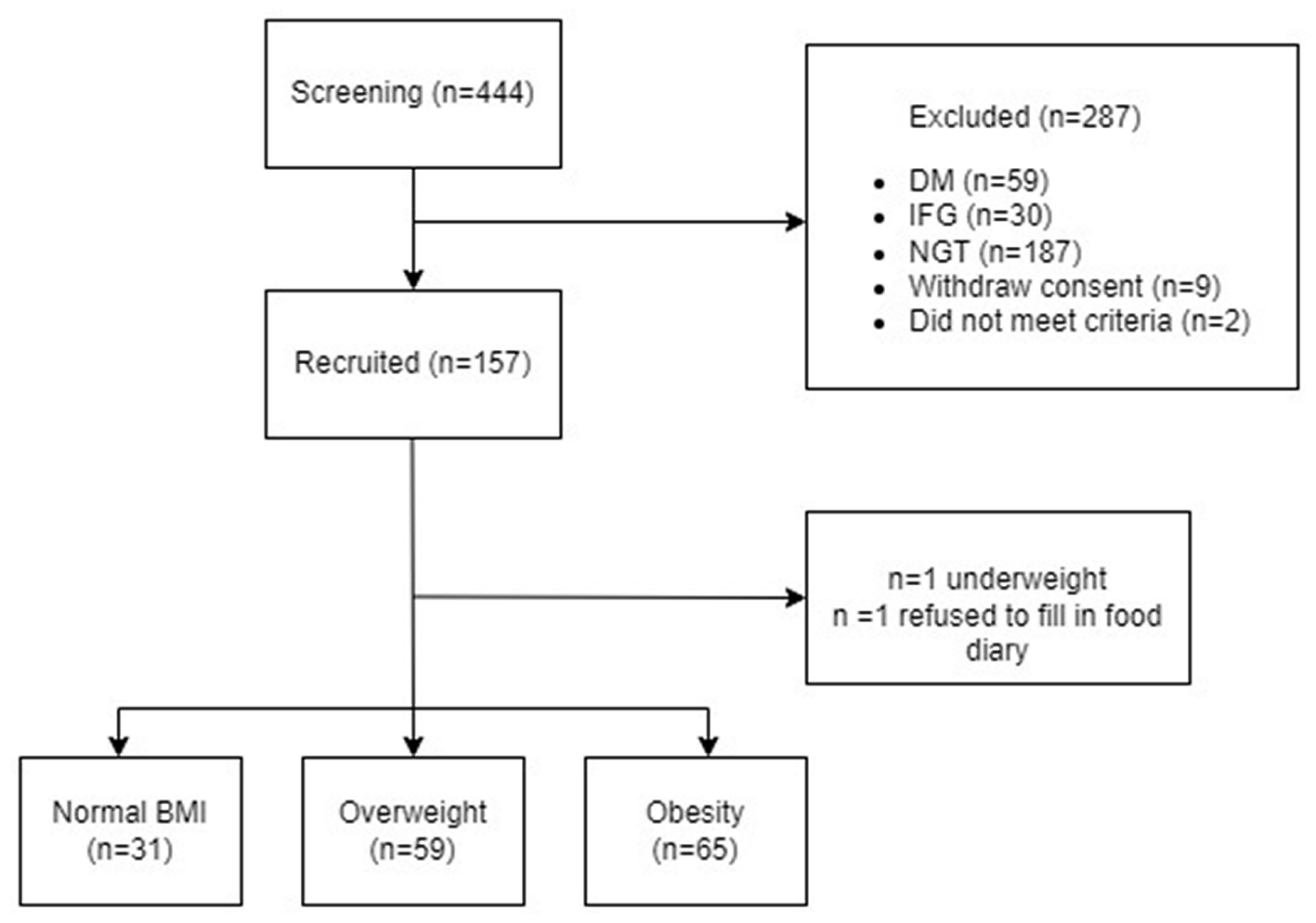
Flow chart of the study. NGT, normal glucose tolerance; IGT, Impaired glucose tolerance; DM, Diabetes mellitus.

A total of 155 individuals with IGT were in included the present analysis (Figure 1). The median age was 59 (53–62) years old with a BMI of 26 (24–29) kg/m^2^ and 58% were female. Table 1 shows the characteristics of the study cohort. 51% of the study cohort had hypertension and 57% had dyslipidaemia. There were no significant differences in cardiovascular diseases, systolic and diastolic blood pressure, heart rate, total cholesterol, and LDL-c amongst different BMI categories (Table 1). IGT participants with obesity had higher plasma triglyceride levels and lower HDL-c when compared to overweight and normal-weight individuals (*p* < 0.0001).

**Table 1:** Characteristics of the study cohort.

Characteristic	Normal weight (*n* = 31) >18 and <23 kg/m^2^	Overweight (*n* = 59) 23–26.9 kg/m^2^	Obesity (*n* = 65) ≥ 27 kg/m^2^
Age, (years)*	58.2 ± 6.0	58.8 ± 6.3	54.9 ± 8.3
Female, (%)	20 ± 64.5	32 ± 54.2	39 ± 59.1
Body mass Index, (kg/m^2^)**	21.5 ± 1.0	25.4 ± 1.2	30.3 ± 2.93
Body fat, (%)**	25.2 ± 6.3	29.3 ± 6.4	36.6 ± 8.8
Hypertension, number (%)	13 ± 41.9	27 ± 45.8	39 ± 59.1
Dyslipidaemia, number (%)	14 ± 45.2	35 ± 59.3	39 ± 59.1
Family history of diabetes, number (%)	18 ± 58.1	41 ± 69.5	32 ± 49.2
Systolic blood pressure, (mm Hg)	129 ± 17.8	133 ± 14.9	135 ± 17.1
Diastolic blood pressure, (mm Hg)	80.1 ± 9.0	84.6 ± 10.0	84.3 ± 11.1
Pluses (beats per min)	71.2 ± 9.5	72.4 ± 10.8	73.5 ± 10.4
Fasting glucose, (mmol/L)	5.3 ± 0.50	5.4 ± 0.52	5.4 ± 0.53
2 h plasma glucose, (mmol/L)	8.1 ± 1.3	8.5 ± 1.5	8.5 ± 1.4
Fasting C-peptide, (pmol/L)**	392 ± 155	602 ± 221	785 ± 283
2 h plasma C-peptide, (pmol/L)**	2,572 ± 757	3,134 ± 1,056	3,478 ± 932
HOMA2-IR**	0.87 ± 0.33	1.36 ± 0.51	1.76 ± 0.64
Total Cholesterol, mmol/L	5.1 ± 1.0	4.8 ± 1.0	4.9 ± 1.0
HDL-C, mmol/L**	1.5 ± 0.39	1.4 ± 0.31	1.2 ± 0.27
LDL-C, mmol/L	3.1 ± 0.85	2.8 ± 0.89	3.0 ± 0.87
Triglycerides, mmol/L**	1.0 ± 0.45	1.3 ± 0.54	1.5 ± 0.88
Light (min/week)	240 (95, 435)	210 (130, 420)	175 (77.5, 338)
Moderate (min/week)	45 (0, 142)	30 (0, 142)	0 (0, 82.5)
Vigorous (min/week)	0 (0, 67.5)	0 (0, 7.5)	0 (0, 22.5)
Sedentary (min/day)	300 (180, 390)	300 (180, 420)	360 (240, 480)

Data were shown as Mean ± SD or median (interquartile range 25th, 75th). Mean ± SD, median (interquartile range), *p = 0.05 and **p ≤ 0.001.

### Association between mineral intake, and body compositions

Individuals with obesity had the lowest consumption of iron, magnesium, and potassium (median 10.6 mg, 241 mg, and 2061 mg) compared with individuals who were overweight (11.9 mg, 282 mg, and 2,284 mg) and normal weight (14.4 mg, 314 mg, and 2,252 mg), (*p* = 0.008, <0.0001 and 0.013). There were no significant differences in other dietary minerals amongst the groups (Table 2). Body weight was positively correlated with the intake of sodium (*r* = 0.16, *p* = 0.040). Body fat was negatively correlated with calcium (*r* = −0.24, *p* = 0.003), chromium (*r* = −0.27, *p* = 0.001), iron (*r* = −0.31, *p* < 0.001), magnesium (*r* = −0.40, *p* < 0.001), phosphorus (*r* = −0.39, *p* < 0.001), potassium (*r* = −0.35, *p* < 0.001), selenium (*r* = −0.27, *p* = 0.001), and sodium (*r* = −0.23, *p* = 0.004) (Table 3). A higher intake of sodium and a ratio of sodium-potassium were correlated to an increase in body weight (*r* = 0.165, *p* = 0.040, and *r* = 0.172, *p* = 0.031).

**Table 2:** Median consumption of macronutrients and minerals by BMI categories.

	Normal (*n* = 31) BMI ≥ 18 and < 23 kg/m^2^	Overweight (*n* = 59) BMI 23–26.9 kg/m^2^	Obesity (*n* = 65) BMI ≥ 27 kg/m^2^
Energy, kcal/day	1774 (1,596, 2,113)	2032 (1802, 2,383)	1858 (1,580, 2,118)
Protein, g/day	91.8 (75.7, 110)	90.8 (82.6, 115)	87 (70.5, 107)
Fat, g/day	74.8 (56.5, 89.2)	81.1 (61.4, 97.9)	77.6 (61.7, 94.0)
Carbohydrates, g/day	195 (152, 232)	204 (159, 250)	199 (161, 243)
Sugar, g/day	38.8 (30.9, 57.3)	40.7 (28.8, 55.2)	40.2 (23.8, 66.1)
Fibre, g/day**	16.7 (13.7, 23.1)	13.5 (10.3, 17.5)	8.8 (6.5, 13.3)
Calcium (mg)	617 (497, 863)	545 (434, 737)	539 (382, 755)
Chromium (mcg)	2.7 (1.9, 5.6)	2.7 (1.9, 3.7)	19 (1.5, 3.5)
Iron (mg)*	13.2 (10.0, 18.6)	10.5 (8.0, 14.5)	10.3 (6.9, 13.3)
Magnesium (mg)**	313 (243, 368)	273 (221, 335)	224 (182, 282)
Phosphorous (mg)	1,158 (898, 1,381)	1,094 (934, 1,342)	999 (824, 1,265)
Potassium (mg)*	2,295 (1833, 3,037)	2,204 (1720, 2,650)	1980 (1,564, 2,365)
Selenium (mcg)	96.6 (73.7, 122)	91.1 (75.1, 110)	86.7 (65.2, 103)
Sodium (mg)	3,531 (2,414, 3,893)	3,754 (3,339, 4,373)	3,677 (2,881, 4,197)
Sodium-potassium ratio*	1.38 (1.07, 1.91)	1.81 (1.29, 2.18)	1.75 (1.26, 2.18)

Median (interquartile range), *p = 0.05 and **p ≤ 0.001.

**Table 3:** Spearmen correlations between minerals and metabolic parameters.

	Calcium (mg)	Chromium (mcg)	Iron (mg)	Magnesium (mg)	Phosphorus (mg)	Potassium (mg)	Selenium (mcg)	Sodium (mg)	Sodium/potassium
Body weight, (kg)	*r* = −0.02, *p* = 0.822	*r* = 0.09, *p* = 0.283	*r* = −0.09, *p* = 0.272	*r* = −0.09, *p* = 0.254	*r* = 0.08, *p* = 0.300	*r* = −0.10, *p* = 0.218	*r* = 0.02, *p* = 0.763	***r* = 0.17, *p* = 0.040**	***r* = 0.17, *p* = 0.031**
BMI, (kg/m^2^)	*r* = −0.10, *p* = 0.213	*r* = −0.11, *p* = 0.156	***r* = −0.23, *p* = 0.005**	***r* = −0.28, *p* = 0.001**	*r* = −0.12, *p* = 0.128	***r* = −0.20, *p* = 0.012**	*r* = −0.13, *p* = 0.102	*r* = 0.00, *p* = 0.994	*r* = 0.12, *p* = 0.147
Body fat (%)	***r* = −0.24, *p* = 0.003**	***r* = −0.27, *p* = 0.001**	***r* = −0.31, *p* < 0.001**	***r* = −0.40, *p* < 0.001**	***r* = −0.39, *p* < 0.001**	***r* = −0.35, *p* < 0.001**	***r* = −0.27, *p* = *r*0.001**	***r* = −0.23, *p* = 0.004**	*r* = 0.85, *p* = 0.298
Systolic blood pressure, (mm Hg)	*r* = −0.04, *p* = 0.590	***r* = −0.18, *p* = 0.028**	*r* = −0.12, *p* = 0.140	*r* = −0.15, *p* = 0.054	*r* = −0.13, *p* = 0.097	*r* = −0.14, *p* = 0.092	*r* = −0.12, *p* = 0.123	*r* = 0.05, *p* = 0.560	*r* = 0.13, *p* = 0.107
Diastolic blood pressure, (mm Hg)	*r* = −0.06, *p* = 0.468	*r* = −0.05, *p* = 0.560	*r* = −0.05, *p* = 0.521	*r* = −0.11, *p* = 0.182	*r* = −0.03, *p* = 0.741	*r* = −0.13, *p* = 0.118	*r* = −0.02, *p* = 0.784	*r* = 0.09, *p* = 0.282	*r* = 0.15, *p* = 0.062
Heart rate (beats per min)	*r* = −0.12, *p* = 0.128	*r* = −0.09, *p* = 0.247	*r* = −0.10, *p* = 0.215	*r* = −0.11, *p* = 0.190	*r* = −0.14 m *p* = 0.094	***r* = −0.19, *p* = 0.016**	*r* = −0.11, *p* = 0.158	*r* = 0.05, *p* = 0.519	***r* = 0.18, *p* = 0.025**
Fasting glucose, (mmol/L)	*r* = 0.00, *p* = 0.999	*r* = −0.07, *p* = 0.375	*r* = 0.08, *p* = 0.305	*r* = 0.04, *p* = 0.619	*r* = 0.10, *p* = 0.196	*r* = 0.00, *p* = 0.970	*r* = 0.04, *p* = 0.598	*r* = 0.10, *p* = 0.228	*r* = 0.07, *p* = 0.059
2 h plasma glucose, (mmol/L)	*r* = −0.02, *p* = 0.796	***r* = −0.19, *p* = 0.019**	*r* = 0.01, *p* = 0.872	*r* = −0.07, *p* = 0.401	*r* = −0.07, *p* = 0.393	*r* = −0.03, *p* = 0.687	*r* = 0.02, *p* = 0.846	*r* = 0.07, *p* = 0.410	*r* = 0.05, *p* = 0.508
Fasting C-peptide, (pmol/L)	*r* = −0.14, *p* = 0.187	*r* = 0.08, *p* = 0.440	*r* = −0.10, *p* = 0.320	*r* = −0.16, *p* = 0.114	*r* = −0.02, *p* = 0.875	*r* = −0.13, *p* = 0.213	*r* = −0.12, *p* = 0.260	*r* = 0.12, 0.239	*r* = 0.19, *p* = 0.059
2 h plasma C-peptide, (pmol/L)	*r* = −0.10, *p* = 0.333	*r* = −0.11, *p* = 0.297	*r* = −0.06, *p* = 0.541	*r* = −0.18, *p* = 0.085	*r* = −0.08, *p* = 0.457	*r* = −0.14, *p* = 0.175	***r* = −0.22, *p* = 0.032**	*r* = 0.05, *p* = 0.595	*r* = 0.20, *p* = 0.051
Total Cholesterol, mmol/L	*r* = 0.02, *p* = 0.791	*r* = −0.08, *p* = 0.347	*r* = 0.04, *p* = 0.612	*r* = 0.04, *p* = 0.633	*r* = −0.02, *p* = 0.789	*r* = 0.07, *p* = 0.372	*r* = 0.03, *p* = 0.682	*r* = 0.08, *p* = 0.293	*r* = 0.27, *p* = 0.735
HDL-C, mmol/L	*r* = 0.05, *p* = 0.543	*r* = −0.11, *p* = 0.178	*r* = −0.07, *p* = 0.400	*r* = 0.02, *p* = 0.820	*r* = −0.09, *p* = 0.264	*r* = 0.06, *p* = 0.468	*r* = −0.11, *p* = 0.164	*r* = −0.15, *p* = 0.070	***r* = − 0.16, *p* = 0.044**
LDL-C, mmol/L	*r* = 0.02, *p* = 0.765	*r* = −0.04, *p* = 0.584	*r* = 0.07, *p* = 0.405	*r* = 0.05, *p* = 0.548	*r* = 0.02, *p* = 0.818	*r* = 0.08, *p* = 0.335	*r* = 0.10, *p* = 0.207	*r* = 0.12, *p* = 0.135	*r* = 0.04, *p* = 0.656
Triglycerides, mmol/L	*r* = −0.07, *p* = 0.391	*r* = −0.03, *p* = 0.669	*r* = 0.05, *p* = 0.522	*r* = −0.04, *p* = 0.586	*r* = 0.01, *p* = 0.861	*r* = −0.05, *p* = 0.514	*r* = 0.00, *p* = 0.977	***r* = 0.17, *p* = 0.036**	***r* = 0.19, *p* = 0.017**
HOMA-IR	*r* = −0.10, *p* = 0.333	*r* = 0.74, *p* = 0.470	*r* = −0.91, *p* = 0.373	*r* = −0.15, *p* = 0.373	*r* = −0.01, *p* = 0.941	*r* = −0.12, *p* = 0.228	*r* = −0.11, *p* = 0.291	*r* = 0.14, *p* = 0.184	***r* = 0.20, *p* = 0.048**
HOMA-B	*r* = −0.15, *p* = 0.137	*r* = 0.13, *p* = 0.209	*r* = −0.13, *p* = 0.207	*r* = −0.20, *p* = 0.051	*r* = −0.08, *p* = 0.450	*r* = −0.13, *p* = 0.205	*r* = −0.12, *p* = 0.235	*r* = 0.01, *p* = 0.895	*r* = 0.12, *p* = 0.249

BMI, body mass index; HOMA2-IR, Homeostatic Model Assessment for Insulin Resistance; Sodium-potassium ratio, the amount of sodium is divided by the amount of potassium in the diet. Bold values are *p*-value less than 0.05.

After the exclusion of those subjects (*n* = 8) who were regularly taking minerals supplementation or Chinese medicine, dietary minerals were still significantly correlated with body fat. It was negatively correlated with calcium (*r* = −0.26, *p* = 0.002), chromium (*r* = −0.29, *p* < 0.0001), iron (*r* = −0.31, *p* < 0.001), magnesium (*r* = −0.40, *p* < 0.001), phosphorus (*r* = −0.41, *p* < 0.001), potassium (*r* = −0.34, *p* < 0.001), selenium (*r* = −0.30, *p* < 0.001), and sodium (*r* = −0.22, *p* = 0.009).

In the multivariate linear regression, there was a significant association between iron and body fat following the adjustment of age and gender (Beta coefficient = −0.277, *p* = 0.006). The association persisted following adjustment after macronutrient intake and fibre in model 1 (Beta coefficient = −0.261, *p* = 0.041) but was inconsistent after the adjustment of physical activity in model 2 (Beta coefficient = −0.179, *p* = 0.167) (Supplementary Table 1). Furthermore, dietary magnesium and potassium were significantly associated with body fat after the adjustment of age and gender (Beta coefficient = −0.020, *p* = 0.001, and −0.002, *p* = 0.001) and macronutrients, fibre (Beta coefficient = −0.024, *p* = 0.003, and −0.003, *p* = 0.002) as well as physical activity (Beta coefficient = −0.030, *p* < 0.0001, and −0.003, *p* = 0.004) (Table 4). The associations between body fat and calcium, chromium, iron, selenium, and sodium were attenuated and became insignificant after the adjustment of macronutrients and physical activity (Supplementary Table 1).

**Table 4:** Multivariate analysis of associations between dietary magnesium, potassium, and body fat.

Dependent variable (Body fat)	Beta coefficient	95% CI	Adjusted *R*^2^
Magnesium			
Base model**	−0.020	[−0.031 to −0.008]	0.485
Model 1*	−0.024	[−0.041 to −0.008]	0.489
Model 2**	−0.030	[−0.046 to −0.013]	0.545
Potassium			
Base model**	−0.002	[−0.004 to −0.001]	0.485
Model 1*	−0.003	[−0.005 to −0.001]	0.492
Model 2*	−0.003	[−0.005 to −0.001]	0.529

Body fat was included as a dependent variable with magnesium, and potassium as independent variables. Base model: adjusted for age, gender; Model 1 = base model + daily intake of total energy (carbohydrates, protein, fats, sugar, and dietary fibre); Model 2 = Model 1 + physical activities (vigorous, moderate, light exercise, and sedentary). *p = 0.005 and **p ≤ 0.001

### Association between mineral intake, lipid profile, insulin secretion, and insulin resistance

There were no associations between iron, magnesium, potassium, and glucose-dependent variables, such as fasting glucose, 2 h postprandial glucose, fasting C-peptide, and HOMA-IR (Table 3). Higher consumption of sodium was correlated with higher plasma triglycerides (*r* = 0.168, *p* = 0.036). A higher ratio of sodium-potassium was correlated with an increase in triglycerides (*r* = 0.191, *p* = 0.017), and HOMA-IR (*r* = 0.201, *p* = 0.048) and lower HDL (*r* = −0.162, *p* = 0.044). In the multivariate linear regression, there was no association between sodium and plasma triglycerides after the adjustment of age and gender in the base model. However, the sodium-potassium ratio was positively associated with HOMA-IR, (Beta coefficient = 0.271, *p* = 0.007), but associations attenuated after the adjustment for macronutrients and fibre (Beta coefficient = 0.130, *p* = 0.227) and physical activity (Beta coefficient = 0.031, *p* = 0.821) (Supplementary Table 2).

## Discussion

In this prospective cross-sectional study, our study suggests an inverse relationship between dietary intake of minerals and body fat. Specifically, higher magnesium and potassium dietary intake was significantly associated with lower body fat, independent of age, macronutrients, physical activity, and fibre intake. The association with other minerals such as calcium, chromium, and selenium became insignificant following the adjustment of other factors.

Recent studies indicated the importance of dietary intake of minerals reduction in risk of chronic diseases, such as obesity, cardiovascular diseases, and diabetes (20, 21). In this study, we observed that magnesium and potassium intake were significantly correlated with lower body fat with persistent adjustment of macronutrients, fibre, and physical activity. Another cross-sectional study in elder adults assessed by a 3-day food record also found that dietary magnesium intake was inversely associated with BMI and metabolic syndrome (22). Magnesium is an essential cofactor for enzymes involved in glucose and insulin metabolism (23), but we did not observe any associations between magnesium and glucose-dependent parameters. We observed a low consumption of magnesium in our study cohort. The recommended daily allowance for magnesium intake is 320 mg and 420 mg for adult females and males, but the intake of magnesium in our cohort was lower, only 237 (192–306) mg in females and 279 (233–360) mg in males. Obesity is characterised by an increase in oxidative stress and magnesium plays an important regulator in participating as a cofactor of several enzymes, and maintenance of cell membrane stability (24, 25). It is suggested that magnesium is involved in redox homeostasis of the multifactorial antioxidant protein PARK7/DJ-1 which is in response to cellular oxidative stress that leads to fat accumulation in adipose tissue (26, 27). Moreover, magnesium affects both phosphorylase b kinase activity and glucose transporter protein activity 4 (GLUT4) by releasing glucose-1-phosphate from glycogen and regulating glucose translocation into the cell in the function of glucose metabolic pathways (28, 29). Studies indicated that magnesium supplementation reduced plasma glucose levels, and improved the glycaemic status of people with prediabetes and risk for developing diabetes (30, 31). However, data on dietary mineral data from dietary records in prediabetes are limited in current publications, patients with prediabetes or diabetes may have a largely inadequate intake of several minerals due to malnutrition and metabolic changes (32).

The gradient of potassium is responsible for maintaining cell function and is maintained in large part by the ubiquitous ion channel *via* the sodium-potassium ATPase pump. Actual dietary potassium requirements would vary with genetics, hypertensive status, and dietary sodium intake. Individuals with hypertension are more sensitive to increasing potassium intake than normotensive individuals and gain a greater benefit for individuals who consumed a high sodium diet (33, 34). Therefore, it is suggested that the balance of sodium and potassium is essential to reduce the risk of hypertension in a particularly high-risk population (35). Meta-analyses showed a significant reduction in blood pressure with increasing potassium supplementation (36, 37), but other studies report inconsistent results (38). High dietary sodium intake has been also correlated to high blood pressure, especially in individuals with elevated plasma triglycerides (39). In our study, the intake both dietary potassium is generally lower than that of the recommended Dietary Reference Intakes (DRIs) (2047 mg/day for females and 2,313 mg/day for males in our cohort versus 2,600 mg/day for females to 3,400 mg/day for males in the recommendation of DRI) (40). However, we observed that potassium was negatively correlated to BMI and body fat, the association between dietary potassium and body fat was robust after the adjustment of age, gender, macronutrients, and physical activity. A pooled meta-analysis indicated that adequate potassium intake and a lower urinary sodium-to-potassium ratio had a protective effect on obesity (13). A possible explanation for this could be dietary potassium maintains cell function, particularly in muscle and nerve activity, and potassium has been shown to positively correlate with an increase in muscle mass (41–43). Skeletal muscle mass plays an important role in metabolic regulation and reduces the percentage of body fat accumulation (44). Furthermore, dietary potassium may relate to Aryl hydrocarbon receptor polymorphisms which are reported to induce weight gain, glucose intolerance, and the development of obesity (45). Similar to the studies of magnesium and the risk of diabetes, studies involving potassium depletion showed that low intake of dietary potassium can lead to glucose intolerance because of the loss of intracellular potassium *via* the ATP-sensitive potassium channel. It may affect impaired insulin secretion in most excitable tissues (46–48). Therefore, habitually low intakes of dietary minerals induce changes in biochemical pathways that can increase the risk of the development of diabetes with obesity (49). Generally, a balanced diet is sufficient to supply the required balance of minerals to help support the metabolism. Recent studies indicated that dietary potassium was not associated with serum potassium or hyperkalemia in either non-dialysis-dependent chronic kidney disease (NDD-CKD) or haemodialysis (HD) patients (50, 51). However, supplement over-consumption may be negative to the metabolism, but the related study is limited.

### Strength and limitations

Firstly, some important confounders were not controlled in this study, such as the use of vitamin and minerals supplements as well as traditional Chinese medicines. These could influence our results, however, 0.5% of subjects (*n* = 8) in the cohort had mineral supplementation or traditional Chinese medicine in the last 3 months prior to the screening. There was a significant correlation between body fat and minerals even after the exclusion of those subjects. Secondly, some local Chinese foods, such as dim sum, did not examine the mineral contents that affect the quantity of habitual mineral contents assessment. Thirdly, bearing in mind the limitations of BMI alone in the assessment of body composition, we did not use more sophisticated techniques such as dual-energy absorptiometry that could better investigate the relationships between dietary minerals, body fat, and muscle mass. Fourthly, we have not yet measured body mineral concentrations with the majority of work being centred on the assessment of urinary or serum minerals for a relevant indicator to evaluate the absorption rate of minerals. Furthermore, we examined the intake of habitual minerals from food records which improves the accuracy and agreement compared to other dietary assessment tools (52), such as the food frequency questionnaire (FFQ). We also assessed subjects’ food records by a research dietitian (Leung K) at the dietary counselling and data were inputted by a research nutritionist (He J) and reconfirmed by a senior research nutritionist (Chu NHS) for tripartite confirmation. Finally, our sample size was relatively small and future studies should be extended to a larger multi-centred cohort.

## Conclusion

A balanced diet with an adequate intake of meats, grains, vegetables, and fruits is essential to health. Apart from the quality of macronutrients, the quantity of minerals may help in different metabolism in carbohydrates and lipids that reduce the body fat, blood pressure, and glucose/ insulin response. Further confirmatory studies are needed to explore associations of dietary magnesium and potassium on the development of obesity and metabolic disorders with implications for the design of nutritional interventions.

## Data availability statement

The original contributions presented in the study are included in the article/Supplementary material, further inquiries can be directed to the corresponding authors.

## Ethics statement

The studies involving human participants were reviewed and approved by The Chinese University of Hong Kong (CUHK) and New Territories East Cluster (NTEC) clinical research ethics committee. The patients/participants provided their written informed consent to participate in this study.

## Author contributions

NC and EC conceived the idea of the study. JH, KL, and NC were involved in data collection and supported by RM and JC. JL and NC analysed the data. NC wrote the first draft of the manuscript. All authors contributed to the article and approved the submitted version.

## Funding

This study was supported by the Health and Medical Research Fund Investigator Initiated Research (17180431) and Hong Kong College of Physicians Young Investigators Research grant 2021 to EC.

## Conflict of interest

The authors declare that the research was conducted in the absence of any commercial or financial relationships that could be construed as a potential conflict of interest.

## Publisher’s note

All claims expressed in this article are solely those of the authors and do not necessarily represent those of their affiliated organizations, or those of the publisher, the editors and the reviewers. Any product that may be evaluated in this article, or claim that may be made by its manufacturer, is not guaranteed or endorsed by the publisher.
