# Supplementary material for: Higher dietary magnesium and potassium intake are associated with lower body fat in people with impaired glucose tolerance
Source: Front Nutr. 2023 Apr 17;10:1169705. doi: 10.3389/fnut.2023.1169705 (PMC10150130; doi:10.3389/fnut.2023.1169705)
Supplement: Supplementary file 1 [file Table_1.DOCX]

Supplementary 1. Multivariate analysis of associations between other dietary minerals and body fat.

| Dependent variable (Body Fat) | Beta coefficient | 95% CI | P value | Adjusted R^2^ |
| --- | --- | --- | --- | --- |
| **Calcium** |  |  |  |  |
| Base model | -0.002 | [-0.005 to 0] | 0.089 | 0.454 |
| Model 1 | -0.003 | [-0.007 to 0] | 0.082 | 0.467 |
| Model 2 | -0.003 | [-0.007 to 0.001] | 0.101 | 0.505 |
|  |  |  |  |  |
| **Chromium** |  |  |  |  |
| Base model | -0.226 | [-0.546 to 0.095] | 0.166 | 0.450 |
| Model 1 | -0.214 | [-0.544 to 0.117] | 0.203 | 0.462 |
| Model 2 | -0.208 | [-0.545 to 0.128] | 0.223 | 0.499 |
|  |  |  |  |  |
| **Iron** |  |  |  |  |
| Base model | -0.277 | [-0.473 to -0.081] | **0.006** | 0.471 |
| Model 1 | -0.261 | [-0.512 to -0.010] | **0.041** | 0.472 |
| Model 2 | -0.179 | [-0.433 to 0.076] | 0.167 | 0.501 |
|  |  |  |  |  |
| **Phosphorus** |  |  |  |  |
| Base model | -0.003 | [-0.005 to 0] | **0.039** | 0.459 |
| Model 1 | -0.004 | [-0.008 to 0] | 0.050 | 0.470 |
| Model 2 | -0.006 | [-0.010 to -0.002] | **0.003** | 0.530 |
|  |  |  |  |  |
| Base model | -0.027 | [-0.058 to 0.005] | 0.093 | 0.453 |
| Model 1 | -0.039 | [-0.081 to 0.004] | 0.072 | 0.468 |
| Model 2 | -0.052 | [-0.0969 to -0.009] | **0.019** | 0.517 |
|  |  |  |  |  |
| **Sodium** |  |  |  |  |
| Base model | 0 | [-0.001 to 0.001] | 0.410 | 0.445 |
| Model 1 | -0.001 | [-0.002 to 0.001] | 0.373 | 0.459 |
| Model 2 | -0.001 | [-0.002 to 0.001] | 0.332 | 0.497 |

Body fat were included as dependent variable with calcium, chromium, iron, phosphorus, selenium and sodium as independent variable

Base model: adjusted for age, gender; Model 1 = base model + daily intake of total energy (carbohydrates, protein, fats, sugar and dietary fiber); Model 2= Model 1 + physical activities (vigorous, moderate, light exercise, and sedentary).

Supplementary 2. Multivariate analysis of association between sodium-potassium ratio and HOMA-IR.

| Dependent variable | Beta coefficient | 95% CI | P value | Adjusted R^2^ |
| --- | --- | --- | --- | --- |
| **HOMA-IR & sodium-potassium ratio** |  |  |  |  |
| Base model | 0.271 | [0.076 to 0.466] | **0.007** | 0.094 |
| Model 1 | 0.130 | [- 0.092 to 0.351] | 0.247 | 0.128 |
| Model 2 | 0.031 | [-0.239 to 0.301] | 0.821 | 0.169 |

HOMA-IR was included as dependent variable with sodium-potassium ratio as independent variable

Base model: adjusted for age, gender; Model 1 = base model + daily intake of total energy (carbohydrates, protein, fats, sugar and dietary fiber); Model 2= Model 1 + physical activities (vigorous, moderate, light exercise, and sedentary).
